# Rapidly Progressive Contralateral Internal Carotid Artery Stenosis After COVID-19 Infection in a Down Syndrome Patient With Unilateral Moyamoya Arteriopathy

**DOI:** 10.7759/cureus.56575

**Published:** 2024-03-20

**Authors:** Blake Wittenberg, Megan Ryan, Jessa Hoffman, Timothy Bernard, Joshua Seinfeld, Corbett Wilkinson

**Affiliations:** 1 Neurosurgery, University of Colorado Anschutz Medical Campus, Aurora, USA; 2 Neurosurgery, Rocky Vista University College of Osteopathic Medicine, Parker, USA; 3 Neurology, University of Colorado Anschutz Medical Campus, Aurora, USA

**Keywords:** moyamoya angiopathy, pediatric moyamoya syndrome, trisomy of 21, down syndrome(ds), mechanical thrombectomy (mt), encephaloduroarteriosynangiosis (edas), moyamoya etiology, sars-cov-2 and covid-19

## Abstract

Moyamoya arteriopathy is a condition where chronic, progressive stenosis of large intracranial arteries, primarily of the anterior circulation, results in ischemia and the growth of small, abnormal collateral vessels. There is increasing evidence that infectious pathologies, such as COVID-19, may serve as a sort of trigger, or “second hit," for the development of moyamoya arteriopathy. In this article, we present the case of a 13-year-old female with Down syndrome and unilateral moyamoya arteriopathy who developed contralateral internal carotid artery (ICA) dissection and thrombus in the setting of a positive COVID-19 test and subsequently developed rapidly progressive contralateral ICA and bilateral anterior cerebral artery (ACA) moyamoya-like stenosis. The rapidly progressive contralateral ICA and bilateral ACA moyamoya-like stenosis are likely multifactorial in nature. The contralateral ICA may have had a predisposition for injury and stenosis due to the preexisting moyamoya arteriopathy, making stenosis more likely after COVID-19-induced vascular inflammation and injury as well as after a possible thrombectomy-associated injury. Based on this presentation, patients with moyamoya arteriopathy may be at risk for rapid progression of their moyamoya pathology when exposed to catalysts, including infection, such as COVID-19, and vascular injury, such as thrombectomy-induced injury. In these circumstances, high suspicion and close monitoring are essential for addressing ischemia related to the stenosis before permanent injury.

## Introduction

Moyamoya arteriopathy is a condition in which there is chronic, progressive stenosis of large intracranial arteries, especially those of the anterior circulation. The stenosis leads to the compensatory growth of small, abnormal collateral vessels at the base of the brain. The new net-like vessels appear like a “puff of smoke” on imaging, hence the name moyamoya in Japanese [1]. Moyamoya arteriopathy can be further divided into moyamoya disease, the idiopathic form, which is more common in people of East Asian descent, and moyamoya syndrome, which occurs in association with known conditions, including Down syndrome [2]. The exact etiology of moyamoya remains unknown. There is suspicion that a protein encoded on chromosome 21 may play a role in moyamoya pathology due to the association between Down syndrome and moyamoya [3].

COVID-19 (coronavirus disease of 2019) is an infectious respiratory disease caused by the SARS-CoV-2 virus. It can lead to coagulopathy and vasculitis that are not limited to the lung and may involve cerebral vessels. Vascular pathologies may include disseminated intravascular coagulation (DIC), deep venous thrombosis, pulmonary embolism, large arterial thrombosis, multiorgan venous and arterial thromboses, and Kawasaki-like disease (a coronary artery vasculitis) [4].

There is increasing evidence that infectious pathologies such as COVID-19 may serve as a sort of trigger, or “second hit," for the development of moyamoya arteriopathy. Ghosh et al. discuss a young patient with no known predisposition presenting with moyamoya-like arterial changes and thalamic hemorrhage after COVID-19 [5]. Das et al. found that 64% of COVID-19 patients with pre-existing moyamoya had worsened their neurological symptoms [6]. In addition, in an international series, Beslow et al. reported several cases of COVID-associated moyamoya in pediatric patients, most of whom presented with arteriopathy [7].

In this case report, we present a patient with Down syndrome and unilateral moyamoya arteriopathy who developed contralateral internal carotid artery (ICA) dissection and thrombus in the setting of a positive COVID-19 test and subsequently developed rapidly progressive contralateral ICA and bilateral anterior cerebral artery (ACA) moyamoya-like stenosis. We postulate that there is an association between this patient’s Down syndrome, COVID-19, and progressive moyamoya arteriopathy.

## Case presentation

**Figure 1:**
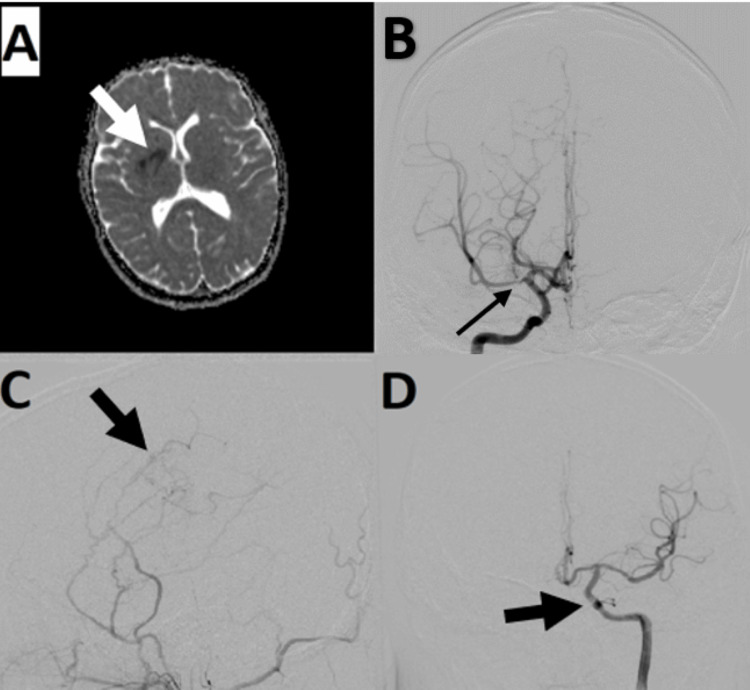
Cranial imaging before the onset of left-sided symptoms 1a: Axial apparent diffusion coefficient (ADC) MRI from 2013 showing hypointensity (arrow) in the right basal ganglia, consistent with an infarct. 1b: AP right ICA angiogram showing narrowing of the right MCA (arrow) seven months after the initial stroke. 1c: Lateral right external carotid artery angiogram showing revascularization (arrow) after EDAS in 2019. 1d: AP left ICA angiogram showing normal caliber of the left ICA in 2013.

A 13-year-old Hispanic female with trisomy 21 and no significant, pertinent family history was initially diagnosed with moyamoya syndrome after a right middle cerebral artery (MCA) territory infarct in 2013 (Figure 1a). Magnetic resonance angiography (MRA) and subsequent cerebral catheter angiography showed right proximal MCA stenosis with dilated lenticulostriate vessels (Figure 1b). She was started on 81 mg aspirin daily and underwent right superficial temporal artery (STA) encephaloduroarteriosynangiosis (EDAS) in 2014 with good postoperative revascularization (Figure 1c). Eventually, she had a complete recovery of her presenting neurologic deficits. She was followed annually clinically and radiographically without any new signs or symptoms of cerebral ischemia. Her right MCA stenosis progressed to complete occlusion, but there was no other moyamoya progression on yearly magnetic resonance angiograms. In 2019, she underwent repeat catheter angiography due to concern for a dural arteriovenous fistula (dAVF) on MRA. Angiography showed continued good revascularization from her EDAS with no dAVF. Importantly, the left internal cerebral artery and subsequent vascular tree had normal caliber and no evidence of early moyamoya (Figure 1d). 

**Figure 2:**
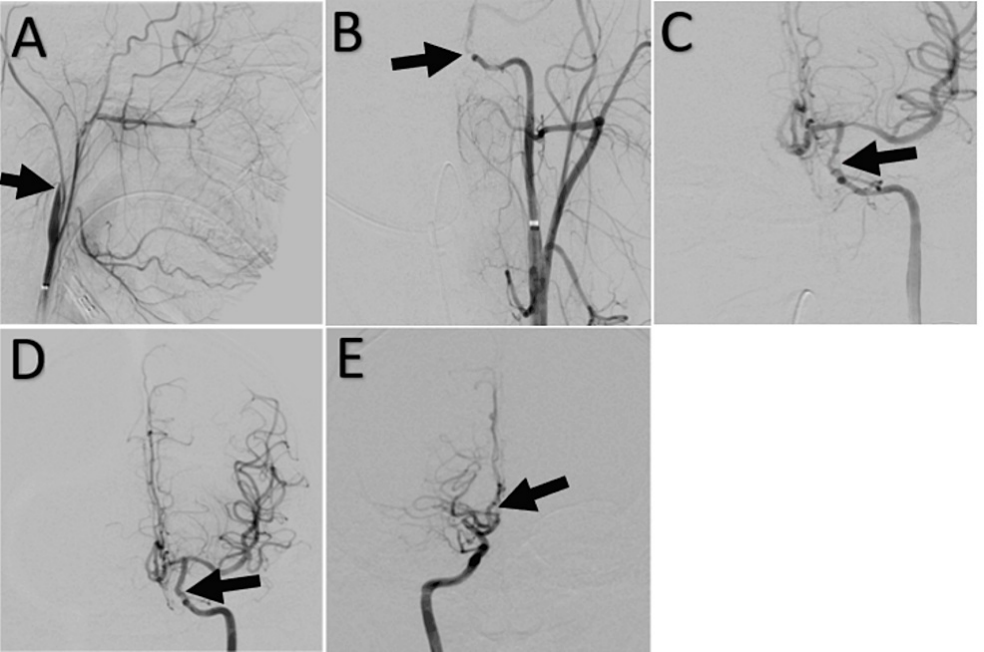
Cranial angiograms during the onset of left-sided symptoms 2a: Lateral left common carotid artery angiogram showing flame-shaped occlusion (arrow) of the cervical ICA just distal to the carotid bifurcation, representing ICA dissection. 2b: AP left ICA angiogram showing a near-occlusive thrombus (arrow) in the proximal cavernous ICA. 2c: AP left ICA angiogram after thrombectomy showing irregularity of the paraclinoid ICA (arrow) representing post-thrombectomy vasospasm. 2d: AP left ICA angiogram showing resolution of paraclinoid ICA vasospasm (arrow) following injection of verapamil.  The left ACA and ICA are normal in caliber. 2e: AP right ICA angiogram showing patent right ACA at time of left ICA thrombectomy (arrow).

In October 2020, she presented with the acute onset of right facial droop, leftward gaze deviation, and right lower extremity weakness. MRA (not shown) revealed a left ICA occlusion. Catheter arteriography showed a flame-shaped occlusion just distal to the origin of the left ICA, consistent with probable dissection (Figure 2a). After navigating past the dissection, a thrombus was encountered in the proximal cavernous ICA (Figure 2b). Using aspiration and a solitaire stent retriever, a successful thrombectomy was performed with complete reperfusion of the affected distribution, TICI3, on the TICI (thrombolysis in cerebral infarction) scale. Post-thrombectomy arteriography revealed vasospasm of the paraclinoid ICA (Figure 2c), which resolved with intraarterial verapamil (Figure 2d). Due to the left cervical ICA dissection, she was started on therapeutic enoxaparin in addition to her baseline aspirin. Further questioning revealed no recent trauma or other injury to explain the ICA dissection. However, during her admission, a nasopharyngeal swab PCR was positive for SARS-CoV-2. She had been asymptomatic before admission and showed no signs or symptoms of COVID infection, other than her presenting dissection or thrombus, during her admission. She had no close contact with anybody known to have COVID-19, but she did have obstructive sleep apnea with visits to the pulmonary clinics a week before presentation. This was thought to be a possible exposure site to COVID-19. The patient had not received any COVID vaccination, as this was not available until December 2020. By discharge, she had returned to her baseline functional and neurologic status. She was discharged on enoxaparin and 81 mg of aspirin per day. The hematologic workup was unremarkable. After six months of dual therapy with enoxaparin and aspirin, the enoxaparin was stopped, and she returned to monotherapy with aspirin.

**Figure 3:**
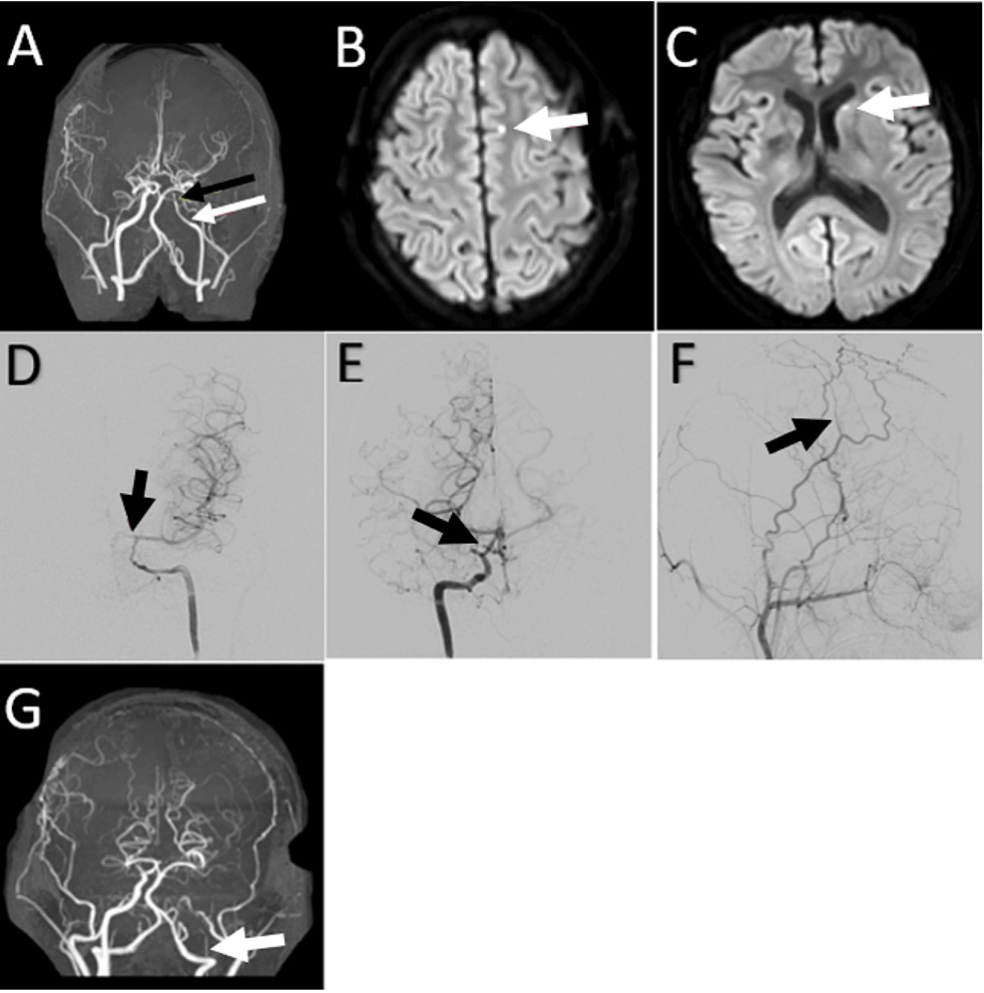
Cranial imaging revealing progressive moyamoya arteriopathy of left-sided vasculature 3a: Coronal MRA MIP from February 2021 showing increased stenosis of the left ICA beginning in the petrous segment (white arrow) and progressing distally (black arrow). 3b: Axial MRI diffusion-weighted images (DWI) obtained three months after left EDAS showing hyperintensity (arrow) in the left superior frontal gyrus consistent with acute ischemia. 3c: Axial DWI MRI obtained three months after left EDAS showing hyperintensity (arrow) in the left caudate head consistent with acute ischemia. 3d: AP left ICA angiogram showing severe left anterior cerebral artery stenosis (arrow). 3e: AP right ICA angiogram showing severe right ACA stenosis (arrow) not seen on previous angiograms (Figure 2e). 3f: Lateral left external carotid artery angiogram showing a patent superficial temporal donor artery (arrow) used for EDAS with no apparent collateral ingrowth to the brain. 3g: Coronal MIP from the most recent MRA showing severe progression of left ICA stenosis into the cervical segment (arrow).

In February 2021, routine follow-up MRA showed new left-terminal ICA stenosis (Figure 3a). She had no new neurologic signs or symptoms, and there were no new signs of ischemia on the MRI. A repeat MRA in March showed further narrowing of the terminal ICA. Based on this progression, she left STA-EDAS in May 2021. MRI/MRA completed three months after surgery showed several punctate areas of acute ischemia in the left superior frontal gyrus and left caudate head (Figures 3b, 3c), as well as bilateral worsened ACA narrowing. Again, there were no neurologic signs or symptoms. A catheter angiogram confirmed the worsened ACA stenosis (Figures 3d, 3e). It also showed a patent left STA donor artery but no apparent collateral ingrowth from the donor artery to the brain (Figure 3f). The patient underwent uncomplicated bifrontal ribbon synangiosis in June 2022. In November 2022, she remained well, but her most recent MRI/MRA shows proximal progression of her left ICA stenosis, with no new ischemic lesions (Figure 3g).

## Discussion

Typical moyamoya progression involves slowly increasing stenosis of the distal ICA as well as the proximal ACA and MCA, leading to transient ischemic attacks and strokes. Our patient’s initial presentation was consistent with moyamoya; however, her later dissection/thrombotic event with subsequent rapidly progressive left ICA stenosis was atypical. In this discussion, we will consider possible etiologies for her findings.

ICA dissection

Cervical artery dissections, involving either the carotid or vertebral arteries, account for only 1-2% of all ischemic strokes but may account for as many as 25% of ischemic strokes in younger individuals. The etiology of cervical artery dissections remains somewhat unclear, but 40% seem to be related to minor trauma. Many believe the cause to be multifactorial, including minor trauma, genetic predisposition, connective tissue diseases, and recent or previous infections [8]. Our patient’s moyamoya syndrome and her COVID both seem likely to have contributed to her spontaneous dissection, as no obvious trauma, major or minor, was reported.

COVID-19 has been implicated as the cause of spontaneous dissections in three previously reported patients, all adults. The first patient was a 39-year-old female with a history of migraines who presented with headaches and neck pain and was found to have bilateral vertebral artery dissections with no instigating trauma but a positive COVID PCR swab on admission [9]. The second was a 38-year-old female with a history of symptomatic COVID one month prior who presented with chest pain radiating to her neck and head and was found to have left common carotid artery dissection, again without any inciting trauma [10]. The third was a 45-year-old female with no history of trauma who presented two weeks after symptomatic COVID with acute-onset occipital headache and left-sided weakness and was found to have left vertebral artery dissection [11]. These three cases, along with ours, may indicate that COVID-19 could rarely cause spontaneous dissections; however, due to the small number of case reports, it remains unknown if asymptomatic COVID-19 infection causes cervical or intracranial dissections. 

ICA thrombus

Our patient’s cavernous carotid thrombus was likely an embolus from her proximal cervical ICA dissection. It was also likely the direct cause of her right-sided symptoms and acute ischemia on imaging rather than hypoperfusion from the cervical ICA dissection. A study by Morel et al. found that, in patients with spontaneous cervical artery dissections, 85% of strokes were attributable to a thromboembolic mechanism rather than hemodynamic compromise related to the dissection [12]. Other causes of ICA thrombus, including cardiogenic or hypercoagulability secondary to COVID-19 infection, are less likely due to the spatial location of the thrombus in relation to the ICA dissection.

ICA stenosis

Possible etiologies for our patient’s rapidly progressing left ICA and bilateral ACA stenosis include an arteriopathy other than moyamoya, possibly COVID-associated vessel injury secondary to the thrombectomy, or progression of moyamoya syndrome. We will discuss these possibilities and their likelihoods below. 

Arteriopathy, including moyamoya arteriopathy, is the most common cause of ischemic stroke in pediatric patients [13]. This differs from adults, in whom embolic and thrombotic etiologies vastly outnumber arteriopathies. Of the ischemic strokes caused by pediatric arteriopathies, only a minority are caused by moyamoya. A multitude of other arteriopathies, including focal cerebral arteriopathy, also known as transient cerebral arteriopathy, are also possible. Differentiating between focal cerebral arteriopathy and moyamoya arteriopathy can be difficult, especially at presentation, as there is significant overlap in their initial presentation. Unlike moyamoya arteriopathy, FCA is characterized by a fluctuating radiologic course of arterial changes over the first days to weeks, followed by a monophasic course for six months without progression and with potential partial or complete resolution [14]. FCA is unlikely to be the underlying cause of our patient’s stenosis. Imaging over two years showed progressive stenosis of the terminal left ICA without any improvement. The rapid progression of cerebral arterial narrowing after a long quiescent period is also atypical of moyamoya.

Thrombectomy-associated vessel wall injury leading to stenosis is a second possible contributing etiology in our patient’s case. No studies have investigated post-thrombectomy stenosis in pediatric patients, likely due to the rarity of thromboses in this population. However, in adult patients, studies have shown a 3.4-8.8% risk of stenosis at three months post-thrombectomy. Most of these cases showed gradual improvement over a 12-month follow-up [15]. Based on the delayed onset of our patient’s left ICA stenosis, which was first identified around four months post-thrombectomy, and her subsequent bilateral ACA stenosis, it is unlikely that thrombectomy was the predominant cause. The progressive worsening of our patient’s left ICA stenosis while on anticoagulation is also inconsistent with the progressively improving stenosis typically seen in adults.

A more likely etiology for our patient’s stenosis is a systemic inflammatory response secondary to COVID-19, when combined with her genetic predisposition to moyamoya. Since the 1980s, inflammation has been posited to be involved in moyamoya pathogenesis [16]. Multiple studies have shown the presence of immune complex mediators within the walls of affected moyamoya vessels. Furthermore, other infections, notably Varicella zoster, have been shown to be associated with arteriopathies, and their antigens have been found within affected vessel walls [14]. COVID-19 has previously been associated with systemic vasculitis and coagulopathies, including Kawasaki-like disease, venous thromboembolism, and large vessel arterial thrombosis [4,15]. A postmortem brain MRI performed on patients who died from COVID-19 but who had shown no premortem clinical signs of cerebrovascular pathology revealed evidence of microvascular abnormalities. Pathologic examination in these patients revealed multifocal cerebrovascular abnormalities, including luminal platelet aggregation and microthrombi, immune complexes with complement activation on platelets and endothelium, and perivascular astrogliosis and microglial nodules [17]. With this pathology seen in patients without underlying cerebrovascular disease, one can imagine the extent of damage that could be seen in patients who do have underlying neurovascular disease, such as moyamoya disease or syndrome. This idea was posited early in the pandemic in a letter to the editor in World Neurosurgery, discussing the possible need to treat and/or surveil patients with moyamoya more aggressively due to their underlying pathology [18].

Taking all the above into account, the most likely etiology of our patient’s stenosis may be multifactorial. The left ICA likely already had a predisposition for injury and stenosis due to the patient’s preexisting moyamoya arteriopathy, making stenosis more likely after COVID-19-induced vascular inflammation and injury as well as after a possible thrombectomy-associated injury. COVID may have triggered the further stenosis in her proximal ACAs. The delayed progression of her left ICA stenosis may be related to the combined effects of her presenting moyamoya syndrome, COVID, and thrombectomy-associated vascular injury.

In actuality, multifactorial etiology may be present in many patients with moyamoya arteriopathy. RNF213 encodes the ring finger protein 213 and is a known moyamoya susceptibility gene. Fujimura et al. studied RNF213 knockout rodents and found no spontaneous moyamoya pathology with just this mutation, although having the mutation put patients at risk for earlier disease onset and more severe moyamoya arteriopathy. RNF213 polymorphism actually increased vascular fragility, which the authors postulated would make the associated vessels more vulnerable to additional stress [19]. The expression of this polymorphism is increased by various infectious and immune markers [20] and is upregulated by lipopolysaccharides, TNFα, and interferon [21]. There is an increasing number of cases of moyamoya arteriopathy seen after infectious etiologies, such as bacterial meningitis and COVID-19, as well as autoimmune conditions, such as Graves’ disease and other thyroid dysfunctions [20]. The above findings add to the suggestion that secondary insults such as infectious (COVID-19) or autoimmune etiologies may be associated with Down syndrome, as there is a predisposition toward autoimmune disease in Down patients, which may be necessary for the development of moyamoya arteriopathy [20,22].

## Conclusions

We present the unusual case of a 13-year-old female with trisomy 21 and known right-sided moyamoya arteriopathy previously treated with EDAS who suffered left ICA dissection and distal occlusion treated by thrombectomy. COVID-19 testing at the time was positive. In the following months, she developed rapidly progressive left ICA stenosis and eventually underwent left EDAS. Subsequent imaging has shown bilateral ACA stenosis as well. We believe that her left ICA and bilateral ACA stenosis are likely multifactorial in origin and are related to her combined underlying moyamoya syndrome, superimposed COVID-19, and possible thrombectomy-associated vascular injury.

In patients with moyamoya arteriopathy, infections, such as COVID-19, and vascular injury, such as from thrombectomy, may be catalysts resulting in the rapid progression of moyamoya pathology. In these circumstances, high suspicion and close monitoring are essential for addressing ischemia resulting from the stenosis before permanent injury.
